# Problematic Peer Functioning in Girls with ADHD: A Systematic Literature Review

**DOI:** 10.1371/journal.pone.0165119

**Published:** 2016-11-21

**Authors:** Francien M. Kok, Yvonne Groen, Anselm B. M. Fuermaier, Oliver Tucha

**Affiliations:** Department of Clinical and Developmental Neuropsychology, University of Groningen, Grote Kruisstraat 2/1, Groningen, The Netherlands; Philipps-Universitat Marburg, GERMANY

## Abstract

**Objective:**

Children with attention deficit hyperactivity disorder (ADHD) experience many peer interaction problems and are at risk of peer rejection and victimisation. Although many studies have investigated problematic peer functioning in children with ADHD, this research has predominantly focused on boys and studies investigating girls are scant. Those studies that did examine girls, often used a male comparison sample, disregarding the inherent gender differences between girls and boys. Previous studies have highlighted this limitation and recommended the need for comparisons between ADHD females and typical females, in order to elucidate the picture of female ADHD with regards to problematic peer functioning. The aim of this literature review was to gain insight into peer functioning difficulties in school-aged girls with ADHD.

**Methods:**

PsychINFO, PubMed, and Web of Knowledge were searched for relevant literature comparing school-aged girls with ADHD to typically developing girls (TDs) in relation to peer functioning. The peer relationship domains were grouped into ‘friendship’, ‘peer status’, ‘social skills/competence’, and ‘peer victimisation and bullying’. In total, thirteen studies were included in the review.

**Results:**

All of the thirteen studies included reported that girls with ADHD, compared to TD girls, demonstrated increased difficulties in the domains of friendship, peer interaction, social skills and functioning, peer victimization and externalising behaviour. Studies consistently showed small to medium effects for lower rates of friendship participation and stability in girls with ADHD relative to TD girls. Higher levels of peer rejection with small to large effect sizes were reported in all studies, which were predicted by girls’ conduct problems. Peer rejection in turn predicted poor social adjustment and a host of problem behaviours. Very high levels of peer victimisation were present in girls with ADHD with large effect sizes. Further, very high levels of social impairment and social skills deficits, with large effect sizes, were found across all studies. Levels of pro-social behaviour varied across studies, but were mostly lower in girls with ADHD, with small to large effect sizes. Overall, social disability was significantly higher among girls with ADHD than among TD girls.

**Conclusion:**

Congruous evidence was found for peer functioning difficulties in the peer relationship domains of friendship, peer status, social skills/competence, and peer victimisation and bullying in girls with ADHD.

## Introduction

Attention deficit hyperactivity disorder (ADHD) is a neurodevelopmental disorder which often persists into adulthood and involves a persistent pattern of inattention and/or hyperactivity-impulsivity that interferes with functioning or development [1]. ADHD affects 5–7% of school age children with a female to male ratio of approximately 1:3 [2,3], and specific gender differences have been found in ADHD symptom presentation and severity [4–6]. Overall, girls score lower on symptom severity of inattention, hyperactivity/impulsivity and total ADHD symptoms than boys [3]. In girls, internalising symptoms and inattentiveness are the more prominent ADHD symptoms, while boys tend to present with externalising symptoms such as impulsiveness and hyperactivity [5,7,8]. Girls also display a different pattern of comorbidity than boys; comorbid internalising disorders (i.e. anxiety and depression) and emotional dysregulation are more prominent in girls [9–13], whereas boys are more likely to present with externalising, disruptive disorders [11,12,14–16]. This co-occurring psychopathology remained stable from childhood to adulthood in girls, but not in boys [6]. Although the symptoms of ADHD in girls are less externalising and more internalising than in boys, and they often present with inattention rather than hyperactivity; the unique difficulties these girls experience are as impairing, persistent and long-lasting as those of their male counterparts [13,17,18]. Furthermore, their impairments may surpass those of boys with ADHD, as has been reported by Swanson, Owens and Hinshaw [19], who found an excessive risk for self-harm and suicide attempts in girls with ADHD. Girls are prescribed medications much less frequently than boys [20–23], although in adulthood medication usage is comparable between women and men [24]. In recent years however, there has been a marked increase in ADHD medication use among girls [24]. Inconsistent findings have been reported on gender differences in response to ADHD medication, with most studies showing no gender differences [8,25] and others showing a slightly more favourable response to medication among boys [8]. Interestingly, young girls are more likely than boys (14% vs 5%) to be treated with antidepressants prior to receiving treatment for ADHD [26], indicating that pharmacotherapy in girls with ADHD tends to be aimed at comorbid conditions such as depression. Taken together, research to date shows that girls with ADHD present with unique, gender-specific issues that require further understanding.

In general, due to the unique presentation of ADHD in girls, they meet fewer ADHD diagnostic criteria as per DSM-5 [7], as such criteria are derived from predominantly male samples. They also tend to receive a diagnosis of ADHD, significantly later than do their male counterparts [7,27]. Further, they are less likely to be referred for assessment and diagnosis in the first place[28]; it has been shown that referral rates for ADHD are much lower for girls than for boys, and also much lower for “gender-typical” girls than for “boyish” girls who present with more externalising symptoms [29]. Due to this referral bias, delayed diagnosis, and lower rates of pharmacotherapy use, ADHD has generally been under-identified and under-treated in girls [18,30]. Added to this mix is the finding that girls with ADHD tend to have better coping skills than boys in this population, which may mask their ADHD symptoms [31,32].

Numerous studies have confirmed that peer functioning is often quite problematic for children with ADHD [33–36]. It is estimated that between 50 and 80% of primary school children with ADHD can be considered peer-rejected, compared to 10–15% of typically developing boys and girls [35,37,38]. In fact, negative peer nominations show the greatest differentiation between children with ADHD and their typically developing peers on sociometric measures [39]. Parents of boys and girls with a history of ADHD report almost three times as many peer problems as those without a history of ADHD [40], and boys and girls with ADHD report greater peer rejection and peer dislike in their own experience as well [34,41–43]. Furthermore, these children have been shown to display increased social withdrawal and isolation [44], and are perceived more negatively on all dimensions of stigma [45,46]. Children with ADHD show much greater peer impairment than children with other psychiatric conditions (i.e. depression, anxiety, learning problems, conduct problems) without ADHD [41,42]. These peer problems are not surprising; after all, hyperactivity and impulsiveness are often associated with negative behaviours such as oppositionality, non-compliance and defiance, which are likely to limit the opportunity for social learning [47]. Children with ADHD may therefore experience less positive peer interactions, and may develop less prosocial skills, leading to decreased quality of peer interactions [48]. Furthermore, the inattentiveness often associated with ADHD may lead to lost opportunities for social learning [34], and it has been proposed that a vicious cycle exists, in which inattentive symptoms predict peers impairment, which in turn leads to increases in both inattention and hyperactivity/impulsivity [49]. Peer functioning is therefore a particularly relevant topic of study in ADHD.

There are several reasons why problematic peer functioning has a major impact on girls with ADHD. First, as girls in general usually have tighter and more intimate social networks [50–53], and as the peer relationships of girls involve higher peer attachment [51–54], disruption to such relationships may impact more negatively on girls than on boys. Second, low self-esteem is more prominent in girls with ADHD relative to typically developing (TD) girls as well as to boys with and without ADHD [55]. Third, children typically tolerate higher levels of ADHD symptoms in boys than in girls [56], and many typical ADHD symptoms are considered more deviant for girls relative to boys [57]. This may be explained by gender expectations of how girls are supposed to behave [58]. Girls with ADHD therefore may stand out from their peers to a higher extent than do boys. Further, a recent study investigating the pathway from ADHD symptoms to depression, found that in girls, the effect of ADHD symptoms on depression was mediated by peer dislike (7%) and victimisation (3%) [59]. Interestingly, this effect was not found in boys. Difficulties in peer functioning may therefore have more severe consequences for girls than for boys.

Due to these gender-specific problems, this review examines problematic peer functioning of girls with ADHD as compared to TD girls rather than to boys with ADHD. After all, the inherent gender differences between girls and boys without ADHD can be considered as different baselines from which to draw comparisons. Girls have different social (interaction) styles than boys and a comparison between girls and boys with ADHD will not give us the female specific information we are looking for. However, in the history of ADHD research, females with ADHD have rarely been compared to females without ADHD. Ohan and Johnston [60] stated that an important limitation of previous research is the use of male comparison samples, rather than comparing girls with ADHD to TD girls. They recommended that future comparisons should be made between ADHD females and typical females across studies, in order to understand the specific needs to women with this disorder. The present review therefore aims to examine data on all-female ADHD samples, to elucidate the picture of female ADHD with regards to problematic peer functioning in this group.

The present literature review will (1) identify the domains in which school-aged girls with ADHD experience problematic peer functioning, (2) describe to what extent these girls differ from TD girls on the identified aspects of peer interaction problems, and (3) identify potential risk factors and protective factors for peer functioning, and describe their influence.

## Method

**Fig 1:**
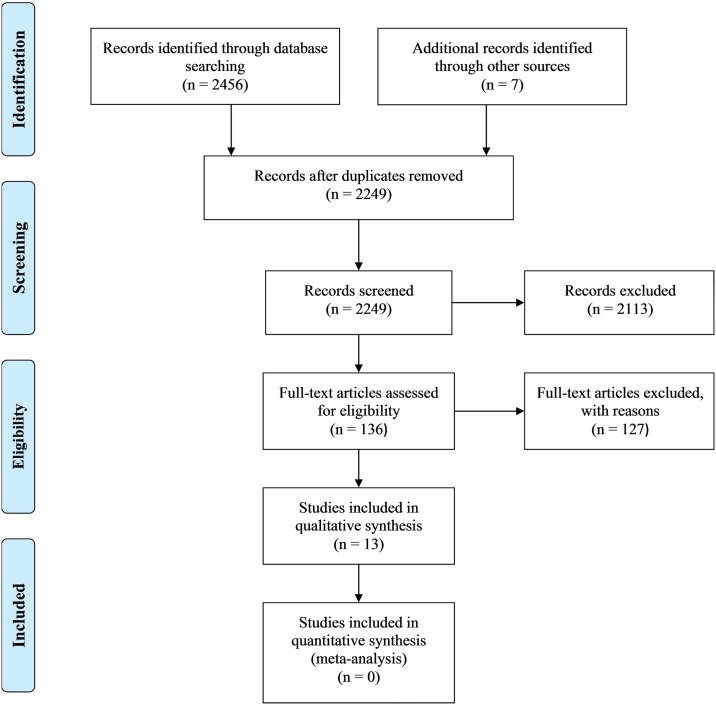
PRISMA 2009 Flow Diagram.

This systematic literature review was performed conforming to the guidelines of Preferred Reporting Items for Systematic Reviews and Meta-Analyses (PRISMA) (see S1 Table for a checklist of the PRISMA guidelines for this study). Several procedures were used to identify potential studies for this review. First, the literature was searched electronically in PsychINFO, PubMed, and Web of Knowledge including all of the available literature up until the date of April 1, 2015. The primary keywords ‘ADHD’ and ‘attention deficit hyperactivity disorder’ were combined with the keywords ‘girls’, ‘female’ and ‘gender’ as well as with keywords related to peer interaction difficulties; such as ‘peer interaction’, ‘social dysfunctioning’, ‘peer relationships’, ‘peer functioning’, ‘peer rejection’, and ‘social isolation’. In order to be included in this meta-analytic review, studies had to meet the following inclusion criteria: (a) the study was written in English; (b) the inclusion of both a sample of girls with ADHD and a sample of comparison girls; (c) the ADHD sample was formally diagnosed with this disorder; (d) the study included girls described as school-age (6–18 years old) only; and (e) the variables measured included at least one peer interaction variable. Finally, reference lists of relevant studies included in the present review were used to locate additional studies. Initial search yielded 2456 records; after removal of duplicates, screening and exclusion of irrelevant articles, 13 studies were included. Most articles were excluded for not including both a sample of girls with ADHD and a comparison sample typically developing girls. Some articles were excluded for including a sample of adult women. After the completion of the search, thirteen studies were included in the review (see Fig 1 for a flow diagram of this process).

Four peer domains emerged from the included studies: friendship, peer status, social skills/competence, and peer victimisation and bullying. These themes are generally consistent with findings of previous research showing that youth with ADHD experience difficulties in these peer functioning areas [36,61,62]. In order to be as complete as possible, a new search was carried out for each variable, in combination with the primary keywords ‘ADHD’ and ‘attention deficit hyperactivity disorder’ and the keywords ‘girls’, ‘female’ and ‘gender’. No additional studies to those already selected were found in this manner.

## Results

The results are categorised by peer functioning variable measured; friendship, peer status, social skills/competence, and peer victimisation and bullying.

Several risk factors (factors that increase the likelihood of impaired peer interaction) as well as protective factors (those that reduce the likelihood of such impairment) were identified in the literature and discussed, as was the impact of psychiatric comorbidities on peer functioning. An overview of the thirteen studies included in this review is presented in S2 Table.

### Friendship

Friendship is a close relationship between two children that is mutual and reciprocal [63].

Two studies investigated friendship in girls with ADHD during a 5-week summer camp for girls, using peer nominations following standard sociometric procedures. Blachman and Hinshaw [64] examined friendship participation, stability and quality and found that girls with ADHD overall were more likely than TD girls to have *no* friends. This did not differ between girls with inattentive-type ADHD (Cohen’s *d* = 0.44) and those with combined-type ADHD (Cohen’s *d* = 0.44). Thirty-two percent of girls with ADHD had no reciprocal friends at the end of camp, relative to 17% of TD girls. Further, 29% of girls with ADHD had *multiple* friends at the end of camp, as compared to 52% of TD girls. The authors concluded that girls with ADHD struggle with making new friends, and, if they do make a friend, they appear less likely to make additional friends than TD girls. Friendship stability was investigated by using peer nominations at various points during the summer camp. No diagnostic group differences were found in friendship stability across week 1 to week 5. However, girls with ADHD Combined type (ADHD-C) were participating in fewer stable friendships than TD girls from week 1 to week 3 (Cohen’s *d* = 0.57); 51% of ADHD-C girls had no stable friendships from the beginning to the middle of camp, relative to 24% of TD girls. The mean number of stable friendships for girls with ADHD Inattentive type (ADHD-I) was in between the ADHD-C and TD means and did not differ significantly from either one. ADHD-I girls were participating in fewer stable friendships than the TD girls from week 3 to week 5 (Cohen’s *d* = 0.68); none of the ADHD-I girls were able to maintain multiple friendships, compared to 29% of TD girls. The mean for the ADHD-C girls fell in between and did not differ significantly from either of the other groups. The authors concluded that ADHD-C girls demonstrate greater initial difficulty maintaining stable friendships whereas ADHD-I girls show more difficulty maintaining multiple friendships over time. Friendship quality was examined by means of self-reports; which showed that the friendships of both ADHD-I and ADHD-C girls contained as much positive relationship features as TD girls, but higher levels of negative relationship features. This included higher levels of conflict, relational aggression within the friendship, and relational aggression to others. These negative relationship features were specific for the ADHD-C girls, with the exception of relational aggression within the friendship, where both ADHD-I and ADHD-C girls had higher levels than TD girls.

Cardoos and Hinshaw [65], using the same summercamp data as Blachman and Hinshaw [64], investigated whether friendship participation could act as a buffer for peer victimisation. They reported that girls with ADHD had significantly lower rates of friendship participation relative to TD girls (Cohen’s *d* = 0.31). It was demonstrated that friendship moderated the association between behavioural risk (externalising and internalising behaviour, social competence) and peer victimisation for the sample as a whole; the presence of at least one friend reduced the risk of victimisation. In addition, girls with ADHD were shown to be similarly protected by the presence of a friendship than were TD girls. Having only friends with a diagnosis of ADHD did not reduce protection relative to having at least one TD friend in both the ADHD and the control group.

To summarise; girls with ADHD were found to have lower rates of friendship participation and stability, which was most pronounced for girls with ADHD-C. Girls with ADHD who had friendships were better liked by their peers than those who did not, independent of whether these friends had a diagnosis of ADHD or not. Friendship quality was lower for girls with ADHD, which was explained by increased levels of negative relationship features within the friendships of these girls, Such negative relationship features which were more pronounced in the girls with ADHD-C compared to ADHD-I. For positive relationship features no difference was found between girls with ADHD and TD girls.

### Peer status

Peer status is the extent to which someone is liked or disliked by their peers [66,67]. Seven studies investigating this variable in girls with ADHD using various measures, including sociometric nominations, were identified. Peer regard was measured in five studies, four of which [64,68–70] demonstrated that girls with ADHD relative to TD girls were less liked, more disliked or had lower peer status. Further, increased levels of peer rejection in girls with ADHD were reported in all studies directly examining this variable. Blachman and Hinshaw [64], using sociometric nomination procedures to calculate positive (peer liking) and negative (peer disliking) nomination scores, further found that lower verbal IQ in girls with ADHD was related to lower peer liking and higher peer disliking during summer camp. Also, the number of friendships during summer camp consistently predicted both peer liking (explaining up to 27% of the variance) and peer disliking; over and above Verbal IQ and diagnostic status. Particularly at the end of camp, the negative relationship between number of friends and peer dislike was significantly stronger for ADHD-C girls than for TD girls. For ADHD-I girls, the pattern was in the same direction but the negative relationship between number of friends and peer dislike was not significantly stronger than for TD girls. The authors suggested that the fewer friends girls with ADHD-C had by the end of camp, the more disliked they were by their peers; whereas for TD girls this effect was only minimal. Thurber, Heller and Hinshaw [69], in their study of social behaviours and peer expectations of girls with ADHD, also reported reduced peer regard in girls with ADHD and moreover noted that this is influenced by the perception of peer responses. Girls with ADHD predicted that their peers would respond negatively to their actions, whereas TD girls predicted positive peer responses. Such perceptions were associated with both observed naturalistic social behaviour and with peer sociometric status such that predictions of negative peer responses contributed significantly to negative peer regard. For girls in the ADHD sample, negative self-reported actions were associated with negative predicted peer responses, and instrumental self-reported actions were associated with both positive and negative predicted peer responses. Overall, girls with ADHD received less positive peer nominations and more negative peer nominations. Mikami and Lorenzi [70] also demonstrated that girls with ADHD had significantly lower peer regard, were impaired in their peer relationships relative to TD girls, and received fewer positive peer nominations (Cohen’s *d* = 0.10). Notably, girls with ADHD and TD girls did not differ on receipt of negative peer nominations. Peer rejection was related to higher levels of problem behaviour, such as defiance, rule-breaking behaviour and aggression. This study also investigated social acceptance through teacher-ratings and peer nominations. Teachers reported the proportion of classroom peers that like and accept the child and the proportion that dislike and reject the child. Girls with ADHD were rated as like/accept less frequent than TD girls (Cohen’s *d* = 1.90), and as dislike/reject more frequent than TD girls (Cohen’s *d* = 1.09). Interestingly, girls with ADHD did not receive less positive nominations than TD girls, and there was a strong negative relationship between conduct problems and positive nominations for both girls with ADHD and TD girls. Finally, this study demonstrated increased levels of peer rejection in their sample of girls with ADHD, which were predicted by externalising behaviours; specifically rule breaking behaviour, observed disobedience, and conflict.

Another non-sociometric study [68], examined the self-reported and teacher-rated popularity of school-aged girls with ADHD on two scales; ‘positive peers’ (popular, smart, athletic) and ‘defiant peers’ (rebellious, good fighter, bad influence). Results showed that girls with ADHD had significantly lower self-reported (Cohen’s *d* = ranging from 0.25 to 0.69) and teacher-reported (Cohen’s *d* ranging from 0.10 to 0.98) popularity ratings. Further, the girls with ADHD were rated as having fewer positive peers (Cohen’s *d* = ranging from 0.13 to 0.89), and more negative peers (Cohen’s *d* = ranging from 0.18 to 0.56), than their comparison counterparts. All results in this study were most pronounced in girls with the inattentive type.

Peer rejection was associated with greater emotional and behavioural problems, and with lower levels of protective variables, in all studies directly examining this variable. More specifically, in the study by Mikami and Hinshaw [71] peer rejection was shown to be related to higher levels of externalising and internalising behaviours, eating pathology and substance use, and to lower levels of academic achievement. Further, peer rejection showed a strong positive correlation with problem behaviours, aggressive behaviour and depressed / anxious behaviour, as well as negative correlations with the hypothesised protective factors popularity with adults and engagement in solitary play. Moreover, popularity with adults and peer rejection predicted depressed / anxious behaviour (marginally significant) and engagement in solitary play and peer rejection predicted aggressive behaviour (marginally significant). The only significant interaction was engagement in solitary play and peer rejection predicting depressed / anxious behaviour. When making a distinction between peer-rejected and peer-accepted girls, popularity with adult staff was more protective in the peer-accepted group; shown by lower levels of depressed / anxious behaviour. Engagement in solitary play was more protective in the peer-rejected group, shown by aggressive as well as depressed / anxious behaviour. The study by Mikami and Hinshaw [17] also found that peer rejection was related to lower levels of self-perceived scholastic competence and engagement in goal-directed play when alone. Girls with ADHD were further found to present with significantly more internalising behaviours than TD girls; both at baseline and at follow-up, and both childhood peer rejection and ADHD diagnosis made significant contributions to greater levels of adolescent internalising behaviours. Both childhood peer rejection and ADHD diagnosis predicted declining adolescent academic achievement. Notably, adolescent externalising and internalising behaviour was no longer predicted by peer rejection and ADHD diagnosis after controlling for childhood levels of these constructs.

No relationship between ADHD in girls and peer regard was found in one sociometric study [72]. This study investigated peer status in adolescent girls with ADHD-I, ADHD-HI, and comparison girls, by means of teacher-ratings. Both ADHD-HI and ADHD-I did not significantly predict negative peer regard. Across groups, negative peer status however predicted school suspensions and expulsions, while peer status at the beginning of summer camp was the only significant predictor of later negative social preference. This study further investigated the impact of internalising behaviours on peer regard by means of girls’ self-reports to investigate such behaviours. Girls with hyperactive-impulsive symptoms were shown to demonstrate higher levels of internalising problems, and higher levels of conduct problems were associated with greater internalising problems. No association was found between internalising symptomatology and peer regard.

In summary; girls with ADHD were more likely to be peer rejected and were less popular than TD girls. However, one study found that although girls with ADHD received fewer positive peer nominations, they received similar negative peer nominations to TD girls. A strong positive relationship was found between conduct problems and teacher-reported peer rejection. Both ADHD and peer rejection predicted poor adjustment. Further, peer rejection was associated with higher levels of problem behaviour, and lower levels of protective variables. When measuring change over time, one study demonstrated that peer rejection and ADHD diagnosis predicted declining adolescent academic achievement.

### Social skills/competence

Social skills/competence refers to possessing the social, emotional, and intellectual skills and behaviours needed to interact positively with others [73]. In total, six studies examing this variable in girls with ADHD were identified. Making use of different scales for social skills/competence, four studies demonstrated lower levels of self- and parent-reports of social competence in girls with ADHD. Greene and colleagues [74] investigated social competence in girls with ADHD through parent ratings. Girls with ADHD scored significantly lower on social competence compared to TD girls as per all three CBCL subscales; ‘activities’, ‘social’ and ‘school’. All girls were further assigned DSM-III-R Global Assessment of Functioning (GAF) scores, which summarizes a child's overall functioning on a scale ranging from 1 to 90; yielding a composite rating of the child's global functioning. Results showed that girls with ADHD had significantly lower GAF scores. Overall social functioning in girls with ADHD was also investigated by means of mother-ratings. It was found that girls with ADHD had significantly lower social functioning than TD girls on multiple subscales, including school behaviour, spare time activities, spare time problems, activity with peers, problems with peers, problems with siblings, and problems with parents. This study additionally examined the variable ‘social disability’, defined as severe social dysfunction, in their sample of girls. Girls with ADHD were found to have a marked overrepresentation of this dysfunction. Results showed that 15% (n = 19) of girls with ADHD qualified as socially disabled versus 1% (n = 1) of TD girls. Similar to social disability, the variable ‘social skills deficits’ was examined by Grskovic & Zentall [75]; who studied the hyperactive, impulsive, social, and emotional characteristics of girls with symptoms of ADHD and TD girls. They used parent-, teacher-, and self-ratings to measure social skills deficits. Their study found that girls with ADHD were rated by teachers, parents, and self as having significantly more social skills deficits than TD girls. Girls’ self-ratings of social skill problems were associated with lower levels of self-esteem.

Sciberras, Ohan and Anderson [76], in their study of bullying and peer victimisation of adolescent girls with ADHD, used girls’ self-ratings to investigate general social problems in this population. They reported that girls with ADHD experienced significantly more social problems (Cohen’s *d* = 1.53 for parent-report and 1.19 for self-report); such as conflict with peers, few social supports and dislike by peers. Further, their social problems were more often clinically significant relative to TD girls, by both parent- and self-report. Similarly, Cardoos & Hinshaw [65], in their study of friendship as protection from peer victimisation in girls with ADHD and TD girls, used parent ratings of social competence. Their study confirmed that girls with ADHD evidenced significantly lower social competence than their comparison counterparts (Cohen’s *d* = -1.37) and that this put them at increased risk of being victimized by their peers. This association existed with both overt and relational victimisation.

Two studies investigated pro-social behaviour, defined as positive social involvement, in girls with ADHD and evidenced contradictory results. Ohan & Johnston [60] studied the impact of ADHD in girl peer interactions using mothers’ and teachers’ ratings as well as observations. In addition they rated the frequency and intensity of pro-social messages through a computerized board game in a controlled laboratory setting. It was shown that, by mother and teacher reports, girls with ADHD were rated as less pro-social than TD girls (Cohen’s *d* = ranging from -0.22 to -5.34). On the laboratory task, girls with ADHD sent less frequent pro-social messages, but the content of the messages they did sent was similar in skills level to those of TD girls. Grskovic & Zentall [75] investigated prosocial behaviour through parent ratings on the Supplementary Descriptive Assessment. Results demonstrated that girls with ADHD were rated similar to TD girls on the Pro-social factor, on eight of the nine parent-rated Pro-social items, and on five of the six self-rated Pro-social items. Further, girls reporting higher levels of pro-social activity reported the highest self-esteem. This pattern occurred in all subgroups of girls; ADHD, LD (Learning Disability) and TD.

In synopsis; increased levels of social impairment, lower levels of social competence, and lower levels of general social functioning were demonstrated in girls with ADHD compared to TD girls. Social disability rates were significantly higher in the ADHD group than in the comparison group. Girls with ADHD also reported more social skills deficits, and these deficits were associated with lower levels of self-esteem. Girls with ADHD not only demonstrated increased levels of general social problems, but also showed more clinically significant social problems. Inconsistent evidence was found for an association between ADHD and pro-social behaviour in girls.

### Peer victimisation and bullying

Peer victimisation refers to being a target of intentional, repeated aggressive behaviour by peers [77], whereas bullying refers to intentional, repeated aggressive behaviour directed at others of lower power or lower perceived social status [78]. Three studies investigating peer victimisation in girls with ADHD were identified, and all found increased levels of this variable in their sample. Cardoos and Hinshaw [65] reported that girls with ADHD were at increased risk of victimisation relative to TD girls (Cohen’s d = 1.29). Based on sociometric peer nominations, they further showed that the presence of a mutual friendship reduced the risk of such victimisation. Further analysis demonstrated that girls with ADHD were no more or less protected against peer victimization by the presence of a friendship than were comparison girls, and having only friends with ADHD was not significantly less protective than having at least one TD friend. Pre-summer internalising behaviour was reported to predict peer victimisation at the summer camps, and, for children with *no* friends, the association between internalising behaviour and victimisation was stronger than for children with one or more friends. In a study on pre-adolescent adjustment in 515 girls with and without ADHD, Elkins and colleagues [68] investigated victimisation by peers through a single-item question and found that girls with ADHD indicated having been bullied much more frequently than their typically developing peers (Cohen’s *d* ranging from 2.54 to 5.20). This pattern was most pronounced in inattentive girls (Cohen’s *d* = 5.20). Further, one study [76] distinguished between overt and relational victimisation in 42 adolescent girls with and without ADHD, using parent- and self-reports. Girls with ADHD manifested higher levels of peer victimisation than TD girls. Specifically, levels of *overt* peer victimisation were significantly higher in the ADHD sample compared to the TD sample, by parent- (Cohen’s *d* = 0.74) and self- (Cohen’s *d* = 1.07) report. Levels of *relational* victimisation were also higher in girls with ADHD, by parent- and self-report, but this result was only significant for parent-report (Cohen’s *d* = 1.06). Further, this study did not demonstrate higher levels of overt and relational bullying in girls with ADHD, by either self- or parent-report.

Overall, in respect of peer victimisation; the studies included in this review demonstrated increased levels of peer victimisation in girls with ADHD. Notably, while overt victimisation was experienced significantly more by both parent- and self-report; relational victimization was only significantly higher in girls with ADHD by parent-report. Although this study did not give an explanation for such a finding, it is plausible that, by the very nature of ADHD, girls may not be aware of relational victimisation to the extent that typically developing girls would be, and that such victimisation may therefore be under-reported by the girls themselves.

### Risk factors and protective factors

All included studies were examined for identified risk and protective factors for the development of problematic peer interaction in girls with ADHD.

#### Risk factors

The main risk factor for problematic peer functioning in girls with ADHD identified was the presence of externalising behaviour (see Table 1). It was established as risk factor in the Cardoos and Hinshaw study [65], who demonstrated that more baseline externalising behaviour was associated with peer problems, in particular peer victimisation, at follow-up. As externalising behaviour is characteristic of the comorbid conditions often seen with ADHD, the impact of this factor is further discussed in detail in section 3.7 on psychiatric comorbidities.

**Table 1:** Summary of risk- and protective factors identified.

Author	Protective factors	Risk factors
**Blachman & Hinshaw (2002)**[64]	Higher friendship status	-
**Cardoos & Hinshaw (2011)**[65]	-	Externalising and internalising behaviour Low social competence
**Lee & Hinshaw (2006)** [72]	-	Low peer status
**Mikami & Hinshaw (2003, 2006)**[17,71]	Self-perceived scholastic competence Engagement in solitary play* Popularity with adult staff*	Peer rejection

* Protective for all girls, both ADHD and TD.

Another identified risk factor was the presence of internalising behaviour in girls with ADHD, which was investigated in two studies. Cardoos and Hinshaw [65], in their study of friendship as protection from peer victimisation in girls with and without ADHD, found a significantly higher mean score on internalising behaviour in girls with ADHD compared to typically developing girls. Pre-summer internalising behaviour was found to predict peer victimisation at the summer camps. Further, for children with no friends, the association between internalising behaviour and victimisation was stronger than for children with one or more friends. Mikami and Hinshaw [17] also found that girls with ADHD presented with significantly more internalising behaviours than TD’s; both at baseline and at follow-up. Further, both childhood peer rejection and ADHD diagnoses made significant contributions to greater levels of adolescent internalising behaviours. Lower peer status/peer rejection were other risk factors identified. Lee and Hinshaw [72] found that baseline lower peer status predicted negative social preference at follow-up five years later, even when initial externalising behaviour was controlled for. Finally, low social competence was identified as risk factor in the study by Cardoos and Hishaw [65], who found that baseline low social competence predicted summercamp peer problems. They also found that the presence of at least one friendship moderated this risk.

#### Protective factors

Higher friendship status was identified as protective factor in one study [64], which demonstrated that the number of mutual friends contributed significantly to the prediction of overall peer liking and disliking at the end of summercamp. For girls with Combined-type ADHD, a lower number of friends by the end of camp was associated with increased peer dislike. However, in comparison girls this association was only minimal. Further, after controlling for peer rejection, popularity with adults predicted lower levels of aggressive behaviour, but not depressed / anxious behaviour. In addition, engagement in solitary play predicted lower levels of depressed / anxious behaviour, but not aggressive behaviour. The only significant interaction of ADHD status and protective factors was that ADHD girls benefited more from solitary play, through lower levels of depressed / anxious behaviour, than TD girls [71]. Popularity with adults had a stronger positive effect in peer-accepted girls than in peer-rejected girls, by lower levels of depressed/anxious behaviour. Engagement in solitary play had a stronger protective effect in the peer-rejected group than in the peer-accepted group as seen in lower levels of both aggressive as well as depressed/anxious behaviour [71].

A summary of identified risk- and protective factors is presented in Table 1.

### Psychiatric comorbidities

Seven studies investigated the impact of comorbidity on peer functioning in their sample; six of these studies demonstrated differences between the comorbid and non-comorbid ADHD group. A summary of these results is presented in Table 2.

**Table 2:** Result summary of studies investigating the influence of comorbidities with ADHD in girls on peer functioning.

Author	N	Mean age (range)	Subtype	Comorbidity measured	Outcome
**Abikoff et al. (2002)**[79]	99 ADHD	8.4 (7–10)	All types	ODD, Anxiety	**Externalising behaviour**
	9 TD	8.4 (7–10)			ADHD + ODD > ADHD ADHD + ODD > ADHD + anxiety ADHD + anxiety = ADHD
**Greene et al. (2001)**[74]	127 ADHD	11.2 (6–18)	All types	ODD, CD, Anxiety	**Social dysfunction at school**
	114 TD	12.2 (6–18)			ADHD + ODD > ADHD ADHD + CD > ADHD
					**Spare time problems, problems with peers**
					ADHD + ODD > ADHD ADHD + CD > ADHD ADHD + anxiety > ADHD
					**Impaired activities with peers**
					ADHD + ODD > ADHD ADHD + anxiety > ADHD
					**Problems with siblings, impaired activities with siblings**
					ADHD + ODD > ADHD
**Grskovic & Zentall (2010)**[75]	20 ADHD	12.8 (range not reported)	Not specified	LD	**Social problems**
					LD = ADHD
	63 TD	10.7 (range not reported)			**Pro-social behaviour**
					LD < ADHD
	19 LD	12.4 (range not reported)			Self-concept*
					LD < ADHD
**Mikami & Lorenzi (2011)**[70]	21 ADHD	8.19 (6–10)	ADHD-C, ADHD-I	CD	**Peer rejection (teacher-report)**
					ADHD + CD > ADHD
	20 TD	8.10 (6–10)			**Positive peer nominations**
					ADHD + CD < ADHD
**Ohan & Johnston (2007)**[60]	22 ADHD + ODD	10.8 (9–12)	ADHD-C, ADHD-I	ODD	**Overt aggression**
					ADHD + ODD > ADHD
					**Confrontationally relational aggression**
	18 ADHD	10.6 (9–12)			ADHD + ODD > ADHD
					**Indirect relational aggression**
	40 TD	10.9 (9–12)			ADHD + ODD = ADHD
					**Pro-social behaviour**
					ADHD + ODD < ADHD
**Sciberras, Ohan & Anderson (2012)**[76]	ADHD 22	15.11 (12–18)	ADHD-C, ADHD-I	ODD	ODD symptoms were associated with increased relational bullying, self-reported overt victimisation, and self-reported social problems. However, as a distinction was not made between ADHD+ODD and ADHD-only; no conclusions should be drawn on the influence of ODD comorbidity.
	TD 20				
**Thurber, Heller & Hinshaw (2002)**[69]	49 ADHD	9.7 (6–12)	ADHD-C, ADHD-I	ODD	**Social goals, social actions & expected peer responses**
	30 TD	9.3 (6–12)			ADHD + ODD = ADHD

Note: TD = typically developing girls, LD = Learning Disability, ODD = Oppositional Defiant Disorder, CD = Conduct Disorder

* As the LD group consisted of both ADHD+LD and LD-only girls, these results do not necessarily show effects of comorbidity

The majority of studies that included girls with both ADHD and ODD/CD, showed impaired social and peer functioning relative to girls with ADHD only. Two studies demonstrated that comorbid ODD and CD diagnosis in girls with ADHD was associated with increased impairment in social functioning relative to girls with ADHD only [70,74] and two studies reported higher levels of externalising behaviour [60,79]. Further, ADHD behaviours adversely impacted social functioning even when comorbidity was controlled for, but the behaviours associated with comorbidity contributed further to this social dysfunction [74]. Three studies investigated the impact of ODD/CD *symptoms* in girls with ADHD; two studies found that such symptoms were associated with increased social impairment, including bullying behaviour and victimization [70,76], but one study did not find such associations [69]. In synopsis, clear evidence was found for impaired social and peer functioning in girls with ADHD and comorbid ODD/CD.

Few studies systematically investigated the impact of other comorbidities. The impact of comorbid anxiety in girls with ADHD was investigated in two studies, however findings were inconsistent. One study compared children with and without ADHD but also included diagnoses of psychopathology, and found that anxiety disorder was associated with impairment on three social dysfunction items [70]. One study compared girls with ADHD and comorbid anxiety with girls with ADHD and comorbid ODD/CD, and with girls with ADHD only and TDs. This study found that comorbid Anxiety did not modify the ADHD effect on any observed behaviour [79]. One study in this review directly compared children with ADHD with and without a comorbid LD and found that LDs in children with ADHD are associated with more social problems, less pro-social behaviour and lower self-concept [75]. However, it is important to note that, as the LD group consisted of both ADHD+LD and LD-only girls, these results do not necessarily show effects of comorbidity. Five studies did not investigate comorbidity in their sample.

## Discussion

The aim of this systematic literature review was to determine in what way girls with ADHD are affected by peer interaction difficulties and to identify risk factors as well as protective factors in this relationship. In total thirteen studies were reviewed that examined peer interaction of female children (10 studies), adolescents (1 study), and both (longitudinal from childhood to adolescence; 2 studies) with ADHD in comparison to TD girls. The studies included in this review incorporated multiple measures of peer functioning, comorbidity, externalising behaviour and protective factors. As these peer functioning dysfunctions were objectified by means of various measurement techniques and informants, namely behavioural observations, peer nominations, and parent-, teacher- and self-reports; confidence in conclusions is considerable.

Congruous evidence for increased peer interaction problems and social dysfunction in girls with ADHD was found. All of the thirteen included studies reported that girls with ADHD, compared to TD girls, demonstrated increased difficulties in the domains of friendship, peer interaction, social skills and functioning, peer victimization and externalising behaviour. Studies consistently showed small to medium effect sizes for lower rates of friendship participation and stability in girls with ADHD relative to TD girls. Higher levels of peer rejection with small to large effect size were reported in all studies, which were predicted by girls’ conduct problems. Peer rejection in turn predicted poor social adjustment and a host of problem behaviours [17,64,68–71]. Very high levels of peer victimisation were present in girls with ADHD with large effect sizes. Further, very high levels of social impairment and social skills deficits, with large effect sizes, were found across all studies. Levels of pro-social behaviour varied across studies, but were mostly lower in girls with ADHD, with small to large effect sizes. Overall, social disability was significantly higher among girls with ADHD than among TD girls. Externalising behaviour, internalizing behaviour, peer status/peer rejection, and low social competence were identified as risk factors. Protective factors noted in the studies included in this review were friendship status, popularity with adult staff, engagement in goal-directed play, and self-perceived scholastic competence. It appears from this review that peer interactions of girls with ADHD are characterized by more negative and aggressive actions than those of TD girls, with medium effect sizes. For instance, one study assessed the social intent of girls with respect to peer interactions using social goal interviews [69]. One of the findings was that girls with ADHD had similar social goals to TD girls, but that they chose aggressive actions in response to peers, predicted negative responses by peers, and acted accordingly. This was in contrast with TD girls, who chose relational actions and predicted positive peer responses. It was suggested that girls with and without ADHD may have a comparable social understanding but a different, defensive style of approach to social actions, characterized by the choice of hostile, aggressive actions as a defence against peers [69].

This negative spiral of problematic peer functioning and the development of social skills has been reported by Mikami and Hinshaw [71], who suggest that peer-rejected girls with ADHD have fewer opportunities for positive social interaction than equally peer-rejected TD girls, and that this overall lack of positive interaction is associated with higher levels of aggression. It has been suggested that peer rejected children and children without reciprocal friends have less exposure to socialisation experiences and less access to social support systems, and that having been rejected by peers and having no reciprocal friends likely increases the risk of being victimised by peers and seeking out other rejected children [65,80]. This in turn could lead to increased exposure to antisocial behaviour and substance use [81]. Such experiences of peer rejection, victimisation, social skills impairment and limited social activities have been shown to be associated with long-term emotional and behavioural difficulties and global impairment [82]. Although behaviour deficits such as lack of pro-social skills, disruptive behaviours and inattention clearly contribute to peer problems, it offers an incomplete picture as it does not consider the contribution of the child’s peer group to her or his problematic peer interaction. Addressing this issue, Mikami and Normand [83] have recently published a conceptual model of peer problems in ADHD, which considers the contribution of the peer group as well as the contribution of the child’s behaviour. They distinguish between three types of peer group influences: social devaluation, exclusionary behaviour and reputational bias. This model may provide a solid framework for future research in girls as well as boys with ADHD.

It has been suggested that social dysfunction in girls with ADHD may be caused by gender-atypical social behaviours. Ohan and Johnston [60] proposed that girls who are overtly aggressive may be at increased risk for future psychological maladjustment and psychopathology, as this type of aggression is more accepted from boys. Because externalising behaviour has been shown to predict peer victimisation, girls with ADHD who in general expose more externalizing behavior may be at greater risk of social adjustment problems during adolescence [68]. This explanation is in line with the previously discussed finding of lower friendship participation of girls with ADHD compared to TD girls. In terms of relational aggression, girls with ADHD sent relationally aggressive messages more frequently than TD girls, but their messages were less severe and they showed less rumour spreading than TD girls. Zalecki and Hinshaw [84] suggest that some forms of relational aggression (e.g. gossiping) involve planning and organizational skills, which are particularly troublesome for children with ADHD. As such, girls with ADHD should show less of this behaviour than TD girls. However, other forms of relational aggression are more sudden, impulsive behaviours; in which case girls with ADHD may show *more* of this behaviour than TD girls. Overall, these externalising and gender-non-normative behaviours often seen in girls with ADHD put them at risk of impairment in many aspects of social functioning.

The problematic social and peer functioning experienced by girls with ADHD may also result from social cognition deficits, such as difficulty perceiving and being attuned to emotions in others, and reasoning about others’ mental state [85]. Inattention may further reduce the ability to be aware of, and tuned in to, subtle social cues and norms. Due to the intimate nature of girls’ social networks, it may be more important for girls to be responsive to such cues than it is for boys, and girls may be expected to be aware of and responsive to these cues to a greater degree than boys, further exacerbating the social dysfunction.

### Strengths and limitations

This review has a number of strengths in exploring peer functioning difficulties in school-aged girls with ADHD. It is unlikely that the outcomes of this review are misrepresented by demographical differences between girls with ADHD and TDs within the studies concerning age, gender, and cognitive ability, because a vast majority of studies controlled for these factors by means of group matching or statistical correction. Further, limited sample size is unlikely to be an issue in this review, with 8 of the 13 studies including an ADHD sample that was bigger than n = 90. Three studies included an ADHD sample size that was smaller than n = 30, which would in general be considered a small sample size. Total sample sizes ranged from 41 to 515 and ADHD sample sizes ranging from 20 to 140.

Despite these strengths, this review has limitations. The findings of this review hold primarily for children, as only one study conducted their investigation in an adolescent sample of girls, and two studies used a longitudinal design. In addition, although according to the Centers for Disease Control and Prevention [86] the percentage of children aged 4–17 taking ADHD medication increased from 4.8% in 2007 to 6.1% in 2011; the vast majority of the studies included in this review used ratings obtained when the girls were not taking medication. The only study that included girls who were taking stimulant / psychotropic medication, found that its use was associated with lower levels of playdate conflict compared to non-medicated girls with ADHD. Findings on the effects of medication use on participant’s peer interaction in the reviewed studies are therefore limited, and no conclusions can be drawn about the effectiveness of medication in improving girls’ peer functioning.

Another limitation of the reviewed studies is that they may suffer from selection bias. Out of the thirteen studies included in this review, four studies use the same sample of girls. In addition, seven of these studies have been prepared by the Stephen Hinshaw workgroup. Selection bias can therefore not be ruled out and it is advisable that independent research groups replicate these findings. Further, the results of boys with ADHD were not included as the focus on this review was on the differences in peer functioning between girls with ADHD and their typically developing counterparts. However, research on gender differences in peer functioning among children with ADHD is scant and shows contradictory results. Some studies found that girls with ADHD were more likely to be reported by teachers as being peer rejected than boys [56,87], whereas other studies reported more parent-reported peer problems in boys relative to girls [5,88]. Other studies have found no gender differences [74,89]. Therefore, further studies are needed to highlight gender differences in social and peer functioning.

## Conclusion

This systematic literature review found congruous evidence for increased peer interaction problems and social dysfunction in girls with ADHD. It highlights the impact of ADHD and related (gender)atypical behaviours across the peer functioning domains friendship, peer status, social skills/competence, and peer victimisation and bullying in girls. Gender-non-normative and externalising behaviours can limit these girls’ opportunities for social learning, leading to delayed development of pro-social skills and a consequent decrease in the quality of their peer interactions. This negative spiral of atypical behaviour and reduced socialization experience exacerbates problematic peer functioning and put girls with ADHD at risk of social maladjustment and psychopathology.

These findings highlight the need for further research as well as careful clinical examination of the different aspects of social dysfunction in girls with ADHD and relevant interventions. It is proposed that effective interventions should be long-term and directly address gender specific peer interaction issues such as negative behaviours and (gender-specific) pro-social skills.

## Supporting Information

S1 File Instruments. (DOCX)

S1 Table PRISMA 2009 Checklist. (DOCX)

S2 Table Summary table of studies included in the present review. (DOCX)
