# Supplementary material for: Problematic Peer Functioning in Girls with ADHD: A Systematic Literature Review
Source: PLoS One. 2016 Nov 21;11(11):e0165119. doi: 10.1371/journal.pone.0165119 (PMC5117588; doi:10.1371/journal.pone.0165119)
Supplement: S1 File — (DOCX) [file pone.0165119.s001.docx]

**Instruments**

1. ACTeRS: ADD-H Comprehensive Teacher's Rating Scale (Ullmann, Sleator & Sprague, 1991).
2. ADHDVRS: ADHD V Rating Scale (DuPaul, 1998).
3. CBCL: Child Behaviour Checklist (Achenbach, 1991).
4. CSBS-T: Children’s Social Behaviour Scale-Teacher form (Crick, 1996).
5. CDI: Children’s Depression Inventory (Kovacs, 1992).
6. COC: Classroom Observation Code (Abikoff, Gittelman & Klein, 1980).
7. EAT: Eating Attitudes Test (Garner, Olmstead, Bohr, & Garfinkel, 1982).
8. EDI-2: Eating Disorders Inventory 2nd Edition (Garner, 1991).
9. DSPS: Dishion Social Preference Scale (Dishion, 1990).
10. FQM: Friendship Qualities Measure (Grotpeter &Crick, 1996).
11. GAF: DSM-II-R Global Assessment of Functioning.
12. Harter Self-Perception Proﬁle for Children (Harter, 1985).
13. K-SADS-E: Schedule of Affective Disorders and Schizophrenia for Children. Epidemiologic Version (Orvaschel & Puig-Antich, 1987).
14. ODDRS: Oppositional Defiant Disorder Rating Scale (Hommerson et al., 2006).
15. Piers-Harris Self-Concept Scale-Popularity Scale (Hur, McGuer & Iacona, 1998).
16. QPQ: Quality of Play Questionnaire (Frankel & Mintz).
17. SAICA: Social Adjustment Inventory for Children and Adolescents (John et al., 1987).
18. Self-Reported Delinquency Scale (Elliott, Huizinga & Ageton, 1985).
19. SEQ: Social Experience Questionnaire (Cullerton-Sen & Crick, 2005).
20. Substance Abuse Questionnaire (Molina, 1995).
21. SUQ: Substance Use Questionnaire (SUQ; Molina & Pelham, 2003).
22. Swanson, Nolan and Pelham Inattention Questionnaire (Swanson, 1992).
23. TRF Scales: Teacher Report Form (Achenbach, 1991).
24. WIAT: Wechsler Individual Achievement Test (Wechsler, 1992).
25. WRAT-III: Wide Range Achievement Test (Wilkinson, 1993).
