# Supplementary material for: Problematic Peer Functioning in Girls with ADHD: A Systematic Literature Review
Source: PLoS One. 2016 Nov 21;11(11):e0165119. doi: 10.1371/journal.pone.0165119 (PMC5117588; doi:10.1371/journal.pone.0165119)
Supplement: S2 Table — (DOCX) [file pone.0165119.s003.docx]

**S2. Table. Summary table of studies included in the present review.**

| **Author** | **N (girls sample)** | **Mean age (range)** | **IQ** | **Med use *n* (%)** | **Subtype**  **(n)** | **Outcome variables measured**  ***Measure* (informant)** | **Outcome, girls with ADHD vs controls** | **Effect Size** |
| --- | --- | --- | --- | --- | --- | --- | --- | --- |
| **Abikoff et al. (2002)** | 99 ADHD  99 NC | 8.4  8.4  (7-10) | Not reported | Not reported | All types | Externalising behaviour  - *Classroom Observation Code* (staff observations) | Externalising behaviour  - Higher rates of non-compliance  - Higher rates of verbal aggression to other children  - Higher rates of solicitation of teacher  Confounding effects  *Comorbidity*  - ODD/CD modified the ADHD effect on  externalising behaviour  - Anxiety did not modify the ADHD effect on any  observed behaviour | - |
| **Blachman & Hinshaw (2002)** | 140 ADHD-C  & ADHD-I  88 NC | 9.5  9.8  9.4  (6-12) | Verbal IQ  ADHD-C: 99.8  ADHD-I: 101.1  NC: 112.7 | Not reported | ADHD-C  ADHD-I | Peer interaction  - *Sociometric nominations* (peer ratings)  Friendship  - *FQM* (self-ratings) | Peer interaction  - More disliked and less liked by peers  Friendship  - Lower rates of friendship participation  - Lower levels of friendship stability during first part  of camp (only significant for ADHD-C girls)  - Lower levels of friendship stability during second  part of camp (only significant for ADHD-I girls)  - Lower levels of friendship participation by the end  of camp was associated with increased peer dislike  Confounding effects  *ADHD subtype*  - Girls with ADHD with more friendships had higher  peer regard, but this was more pronounced in  ADHD-C than in ADHD-I.  - ADHD-I girls had increased levels of relational  aggression within friendships  - ADHD-C girls had increased levels of all negative  relationship features within friendships | ADHD-C vs NC: *d*=0.46  ADHD-I vs NC: *d*= 0.44  ADHD-C vs NC: *d*= 0.57  ADHD-I vs NC: *d*= 0.35  ADHD-C vs NC: *d*= 0.34  ADHD-I vs NC: *d*= 0.68 |
| **Cardoos & Hinshaw (2011)** | 140 ADHD-C  & ADHD-I  88 NC | 9.5  9.8  9.4  (6-12) | Not reported | 27 (19.3%) | ADHD-C  ADHD-I | Externalising behaviour  - *CBCL* (parent ratings)  - *TRF* (teacher ratings)  Social skills and functioning  - *CBCL* (parent ratings)  Friendships  - *Sociometric nominations* (peer ratings)  Peer victimisation  - *Sociometric nominations* (peer ratings) | Externalising behaviour  - Increased levels of externalising behaviour  Social skills and functioning  - Decreased levels of social competence  Friendships  - Low number of friendships  - The number of friendship moderated the impact of  ADHD in terms of internalising behaviour,  externalising behaviour, and social competence.  Peer victimisation  - Increased levels of victimization  - Girls with NC friends were no more or less  protected from victimisation than those with all  ADHD friends | *d*= 2.67  *d*= 1.37  *d*= 0.31  *d*= 1.29 |
| **Elkins et al. (2011)** | 109 ADHD  406 NC | 11.9  11.9  (11) | Full Scale IQ  ADHD-I 96.1  ADHD-HI 101.4  ADHD-C 98.8  NC 102.3 | 33 (30.3%) | ADHD-C  ADHD-I  ADHD-H | Peer victimisation  - *Single item question* (self-ratings)  Peer functioning  *-* *Piers-Harris Self-Concept Scale-Popularity*  *Scale** (self-ratings)  - *Popularity rating* (teacher ratings)  - *Rating on positive peers and deviant peers* (teacher ratings) | Peer victimisation  - Increased levels of victimisation  Peer functioning  - Lower self-rated popularity  - Lower teacher-rated popularity  - Fewer positive peers  - More negative peers  Confounding effects  *ADHD subtype*  - The increased levels of victimisation was most  pronounced in girls with ADHD-I. | ADHD-C vs NC: OR=4.80  ADHD-I vs NC: OR=5.20  ADHD-H vs NC: OR=2.54  ADHD-C vs NC: *d*=-0.25  ADHD-I vs NC: *d*=-0.69  ADHD-H vs NC: *d*=-0.38  ADHD-C vs NC: *d*=-0.10  ADHD-I vs NC: *d*=-0.98  ADHD-H vs NC: *d*=-0.37  ADHD-C vs NC: *d*=-0.13  ADHD-I vs NC: *d*=-0.89  ADHD-H vs NC: *d*=-0.13  ADHD-C vs NC: *d*=0.56  ADHD-I vs NC: *d*=0.18  ADHD-H vs NC: *d*=0.35 |
| **Greene et al.**  **(2001)** | 127 ADHD  114 NC | 11.2  12.2  (6-18) | Not reported | Not reported | All types | Social skills and functioning  - *SAICA* (parent ratings)  - *CBCL* (parent ratings) | Social skills and functioning  - Increased levels of social impairment  - Decreased levels of social competence  - Greater impairment on ‘activity with peers’ and  ‘problems with peers’  - Lower levels of general functioning  - Increased levels of social disability (15% ADHD,  1% NC)  Confounding effects  *Comorbidity*  - ODD was associated with dysfunction at school,  spare-time problems, spare-time activities, problems  with peers, impaired activities with peers, problems  with siblings, impaired activities with siblings and  problems with parents.  - CD was associated with social dysfunction at  school, spare-time problems, and problems with  peers.  - Anxiety Disorder was associated with spare-time  problems and problems with peers, and with  impairment on spare-time activities and activities  with peers | - |
| **Grskovic & Zentall (2010)** | 20 ADHD  63 NC  19 LD | 12.8  10.7  12.4  (range not reported) | Standard IQ  ADHD: 105.6  LD: 113.1  NC: 95 | Not reported | All types | Social skills and functioning  - *ACTeRS* (teacher, parent and self-ratings)  - *ACTeRS* Supplementary Descriptive  Assessment (parent and self-ratings) | Social skills and functioning  - More social skills deficits.  - Girls’ self-ratings of social skill problems were  associated with lower levels of self-esteem  Confounding effects  *Comorbidity*  - LD was associated with more social problems, less  pro-social behaviour and lower self-concept. |  |
| **Lee & Hinshaw (2006)** | 140 ADHD-C  & ADHD-I  88 NC | 9.5  9.8  9.4  (6-12) | Verbal IQ  ADHD-HI: 99.8  ADHD-I: 101.1  NC: 113.1 | 23 (16.4%) | ADHD-C  ADHD-I | **Baseline measures**  Externalising behaviour  - *Observation* (staff observations)  - *Laboratory measure* (self-ratings)  Peer interaction  - *Sociometric nominations* (peer ratings)  **Follow-up measures**  Social skills and functioning  - *DSPS* (teacher ratings) | Peer interaction  - Lower social preference  - Lower peer status  - Higher levels of externalising behaviour  - Negative peer status predicted school suspensions  and expulsions  - Initial peer status predicted negative social  preference  Confounding effects  *ADHD subtype*  - ADHD-HI symptoms predicted adolescent conduct  problems, substance use and internalising  problems.  - ADHD-I symptoms predicted academic  achievement, school suspensions and expulsions |  |
| **Ohan & Johnston (2007)** | 22 ADHD + ODD  18 ADHD only  40 NC | 10.8  10.6  10.9  (9-12) | Not reported | 22 (55%) | ADHD-HI  ADHD-I | Externalising behaviour  - *Parallel version of SCBS-T* (parent ratings)  - *CSBS-T* (teacher ratings)  - *Laboratory paradigm* (self-ratings)  Social skills and functioning  - *Parallel version of SCBS-T* (parent ratings)  - *CSBS-T* (teacher ratings)  - *Laboratory paradigm* (self-ratings) | Externalising behaviour  - Increased levels of overt aggression  - Increased levels of relational aggression, but less  rumour spreading  Social skills and functioning  - Lower levels of pro-social behaviour  - Higher levels of awkward interactions  - Although NC girls were less likely to send  relationally aggressive messages than the other  groups, their relationally aggressive messages were  more intense.  Confounding effects  *ADHD subtype*  - ADHD-HI symptoms predicted overt and relational  aggression  - ADHD-I symptoms predicted relational aggression  *Comorbidity*  - ODD symptoms predicted overt and relational  aggression and less pro-social behaviour. | *d*= 0.77 (Mother-report)  *d*= 2.03 (Lab: mesg. freq.)  *d*= 0.87 (Lab: mesg. Int.)  *d*= 0.82 (Mother-report)  *d*= 1.19 (Teacher-report)  *d*= 0.49 (Lab: mesg. freq.)  *d*= -0.93 (Lab: mesg. int.)  *d*= 0.41 (Lab: social excl.)  *d*= -0.76 (Lab: rumour spr.)  *d*= -0.68 (Mother-report)  *d*= -0.22 (Teacher-report)  *d*= -5.34 (Lab: mesg. freq.)  *d*= -0.33 (Lab: mesg. int.)  *d*= 5.44 (Lab: mesg. freq.)  *d*= 0.70 (Lab: mesg. int.) |
| **Mikami & Hinshaw (2003)** | 91 ADHD  58 NC | 9.5  9.1  (6-12) | Verbal Comprehension  ADHD-C: 100.6  ADHD-I: 102.8  NC: 113.7  Processing Speed  ADHD-C: 100.2  ADHD-I: 99.4  NC: 108.1 | Not reported | ADHD-C  ADHD-I | Peer interaction  - *Sociometric nominations* (peer ratings)  Externalising behaviour  *- CBCL Aggressive behaviour and*  *Delinquent Behaviour narrow-*  *band Scale* (parent ratings)  - *TRF* (teacher ratings)  - *Observation* (staff observations and  observer observations) | Peer interaction  - Higher levels of peer rejection  - Peer rejection related to higher levels of problem  behaviour.  - Peer rejection related to lower levels of protective  variables  Externalising behaviour  - Higher levels of aggressive behaviour  - Popularity with adults predicted lower levels of  aggression  Confounding effects  *ADHD subtype*  - Girls with ADHD-I were less peer-rejected and  displayed lower rates of aggressive behaviour |  |
| **Mikami & Hinshaw (2006)** | Baseline:  140 ADHD-C  & ADHD-I  88 NC  Follow-up:  127 ADHD  82 C | Baseline:  9.5  9.8  9.4  (6-12)  Follow-up:  M not reported  (11-18) |  |  | ADHD-C  ADHD-I | **Baseline measures**  Peer interaction  *- Sociometric nominations* (peer ratings)  - Parent-reports  - *DSPS* (teacher ratings)  Externalising behaviour  - *CBCL* Aggressive behaviour and  Delinquent Behaviour narrow-band Scale.  (parent ratings)  - *TRF* (teacher ratings)  - *Observation* (staff ratings and observer  ratings)  **Follow-up measures**  Externalising behaviour  - *CBCL* (parent ratings)  - *TRF* (teacher ratings)  - *Self-Reported Delinquency Scale*****  (self-ratings) | **Baseline**  Peer interaction  - Increased levels of peer rejection  - Peer rejection associated with problem behaviour  Externalising behaviour  - Increased levels of externalising symptoms  **Adolescent outcomes**  Peer rejection  - Increased levels of peer rejection  - Peer rejection associated with problem behaviour  - Peer rejection related to lower self-perceived  scholastic competence, lower engagement in goal-  directed play when alone, and lower popularity with  adults  - Childhood peer rejection and ADHD diagnosis were  associated with greater levels of internalising and  externalising behaviours, greater eating pathology,  and lower levels of academic achievement.  - Peer rejection and ADHD diagnosis predicted  declining academic achievement into adolescence.  Externalising behaviour  - Higher levels of externalising symptoms |  |
| **Mikami & Lorenzi (2011)** | ADHD 21  NC 20 | 8.19  8.10  (6-10) | Verbal IQ  ADHD 101.67  NC 114.60 | 13 (28.6%) | ADHD-C  ADHD-I | Externalising behaviour  - *TRF-Rule Breaking Behaviour subscale*  (teacher rating)  - *Observation during free play sessions*;  Likert scale (staff observations)  - *QPQ-Conflict Subscale* (parent ratings)  Social skill and functioning  - *DSAS* (teacher ratings)  Peer interaction  - *Sociometric nominations* (peer ratings) | Externalising behaviour  - Higher levels of parent-reported playdate conflict.  Peer interaction  - Higher levels of teacher-reported peer rejection  - Lower levels of teacher-reported peer acceptance  - Fewer positive sociometric nominations received  Confounding effects  *Comorbidity*  - Strong positive relationship between conduct  problems and teacher-reported peer rejection in girls  with ADHD.  - Strong negative relationship between conduct  problems and positive peer nominations. | *d*= 1.19  *d*= 1.09  *d*= -1.90  *d*= 0.10 |
| **Sciberras, Ohan & Anderson (2012)** | ADHD 22  NC 20 | 15.11  15.11  (12-18) | Verbal IQ  ADHD 91.3  NC 91.3 | 16 (72.3%) | ADHD-C  ADHD-I | Peer victimization  *- SEQ* (self- and parent ratings)  Externalising behaviour  - *CSBS* (self- and parent ratings)  Social skills and functioning  - *Youth Self-report (YSR) version of CBCL*  (self-ratings) | Peer victimisation  - Higher levels of overt victimisation  - Higher levels of relational victimisation (parent-  report only)  Social skills and functioning  - Higher levels of social problems  - More clinically significant social problems | *d*= 0.74 (parent-report)  *d*= 1.07 (self-report)  *d*= 1.06  *d*= 1.53 (parent-report)  *d*= 1.19 (self-report) |
| **Thurber, Heller & Hinshaw (2002)** | ADHD 49  C 30 | 9.7  9.3  (6-12) | Not reported | Not reported | ADHD-C  ADHD-I | Social skills and functioning  - *Social goals interviews* (interview)  Peer interaction  - *Sociometric nominations* (peer ratings)  Externalising behaviour  - *Observation* (staff observations) | Social skills and functioning  - Less negotiating actions  Peer interaction  - More positive nominations  - Less negative nominations  - Anticipated more negative peer responses  - Anticipated less positive peer responses  - Aggressive behaviours were associated with  negative peer  Responses in girls with ADHD  - Instrumental behaviours were associated with  positive and negative peer  Responses in girls with ADHD  Externalising behaviour  - More observed physical aggression  - More self-reported aggressive actions to attain goals  - Higher levels of ODD symptomatology |  |

Note: * Hur, McGuer & Iacona (1998) **Swanson (1992) ***Harter (1985) ****Elliott, Huizinga, & Ageton (1985) *****Molina (1995).

**ADHD-C**: Combined-type ADHD, **ADHD-HI**: Hyperactive/Impulsive-type ADHD, **ADHD-I**: Inattentive-type ADHD. **ADHDVRS**: ADHD V Rating Scale (DuPaul, 1996), **CBCL**: Child Behaviour Checklist (Achenbach, 1991), **CSBS-T**: Children’s Social Behaviour Scale-Teacher form (Crick, 1996), **CDI**: Children’s Depression Inventory (Kovacs, 1992), **COC**: Classroom Observation Code (Abikoff & Gittelman, 1985), **EDI-2**: Eating Disorders Inventory 2^nd^ Edition (Garner, 1991), **DSPS**: Dishion Social Preference Scale (Dishion, 1990); **EAT**: Eating Attitudes Test (Garner, Olmstead, Bohr, & Garfinkel,1982), **FQM**: Friendship Qualities Measure (Grotpeter &Crick, 1996**)**, **GAF: DSM-II-R**: Global Assessment of Functioning**, K-SADS-E**: Schedule of Affective Disorders and Schizophrenia for Children. Epidemiologic Version (Orvaschel & Puig-Antich, 1987), **NC**: Normal Controls, **ODD**: Oppositional Defiant Disorder; **ODDRS**: Oppositional Defiant Disorder Rating Scale (Hommerson et al., 2006), **QPQ**: Quality of Play Questionnaire (Frankel & Mintz), **SAICA**: Social Adjustment Inventory for Children and Adolescents (John et al., 1987), **SEQ**: Social Experience Questionnaire (Cullerton-Sen & Crick, 2005), **SUQ**: Substance Use Questionnaire (SUQ; Molina & Pelham, 2003), **TRF Scales**: Teacher-report Form (Achenbach, 1991), **WIAT**: Wechsler Individual Achievement Test (Wechsler, 1992), **WRAT-III**: Wide Range Achievement Test (Wilkinson, 1993).
